# Muscular weakness and muscle wasting in the critically ill

**DOI:** 10.1002/jcsm.12620

**Published:** 2020-09-07

**Authors:** Joerg C. Schefold, Tobias Wollersheim, Julius J. Grunow, Markus M. Luedi, Werner J. Z'Graggen, Steffen Weber‐Carstens

**Affiliations:** ^1^ Department of Intensive Care Medicine Inselspital, Bern University Hospital, University of Bern Bern Switzerland; ^2^ Department of Anesthesiology and Operative Intensive Care Medicine (CCM, CVK) Charité—Universitätsmedizin Berlin, Freie Universität Berlin, Humboldt Universität zu Berlin and Berlin Institute of Health Berlin Germany; ^3^ Berlin Institute of Health (BIH) Berlin Germany; ^4^ Department of Anaesthesiology and Pain Medicine Inselspital, University Hospital Bern, University of Bern Bern Switzerland; ^5^ Department of Neurology and Neurosurgery Inselspital, University Hospital Bern, University of Bern Bern Switzerland

**Keywords:** Critical illness myopathy, Critical illness polyneuropathy, Dysphagia, Swallowing disorder, ICU‐acquired weakness, Sepsis

## Abstract

**Background:**

Muscular weakness and/or muscle wasting is recognized as a key medical problem in critically ill patients on intensive care units (ICUs) worldwide.

**Methods and Results:**

Intensive care unit‐acquired weakness (ICUAW) results from various diseases leading to critical illness and is observed in about 40% [1080/2686 patients, 95% confidence interval (CI): 38–42%] of mixed (medical–surgical) ICU patients. Muscle strength at ICU discharge is directly associated with mortality 5 years after discharge [hazard ratio 0.946, 95% CI: 0.928–0.968 per point increase in Medical Research Council (MRC) scores, *P* = 0.001]. ICUAW serves as umbrella term for the subgroups ‘critical illness myopathy’, ‘critical illness polyneuropathy’, and ‘critical illness polyneuromyopathy’, the latter distinguished using electrophysiology and/or biopsy studies. Diagnosing, studying, and developing treatments for ICUAW among the critically ill seems challenging due to the acuity and severity of the underlying heterogeneous diseases. Ventilator‐induced diaphragmatic dysfunction occurs in up to 80% (*n* = 32/40) of ICUAW patients after mechanical ventilation and mostly results from distinct muscular pathologies, disuse, underlying critical illness, and/or effects imposed directly by mechanical ventilation. Swallowing disorders/dysphagia likely represent an additional (local) neuromuscular dysfunction/ICUAW sequelae and presents in 10.3% (*n* = 96/933) of mixed medical–surgical ICU survivors, with 60.4% (*n* = 58/96) of patients remaining dysphagia positive until hospital discharge. Key independent risk factors for dysphagia following mechanical ventilation are baseline neurological disease [odds ratio (OR) 4.45, 95% CI: 2.74–7.24, *P* < 0.01], emergency admission (OR 2.04, 95% CI: 1.15–3.59, *P* < 0.01), days on mechanical ventilation (OR 1.19, 95% CI: 1.06–1.34, *P* < 0.01), days on renal replacement therapy (OR 1.1, 95% CI: 1–1.23, *P* = 0.03), and disease severity (Acute Physiology and Chronic Health Evaluation II score within first 24 h; OR 1.03, 95% CI: 0.99–1.07, *P* < 0.01). Dysphagia positivity independently predicts 28‐day and 90‐day mortality (90‐day univariate hazard ratio: 3.74; 95% CI, 2.01–6.95; *P* < 0.001) and is associated with a 9.2% excess (all‐cause) mortality rate.

**Conclusions:**

Neuromuscular weakness and muscle wasting is observed in many survivors of critical illness. ICUAW, ventilator‐induced diaphragmatic dysfunction, and dysphagia are associated with complicated and prolonged ICU stay, impaired weaning from mechanical ventilation, impeded rehabilitative measures, and a considerable impact on morbidity and mortality is noted. Future research strategies should further explore underlying pathomechanisms and lead to development of causal treatment strategies.

## Introduction

Reduced muscular force, as in intensive care unit (ICU)‐acquired weakness (ICUAW), is observed in many, if not most, survivors of critical illness following various critical diseases.^1^, ^2^, ^3^, ^4^, ^5^ Clinical consequences of muscular weakness include, for example, impaired mobilization, prolonged bed rest, and extended ICU and/or hospital length of stay. This may induce a ‘vicious cycle’ of (secondary) complications and necessitates repeated and/or intensified medical therapy, which may again result in increased morbidity and mortality.^2^, ^3^, ^4^, ^5^, ^6^ Further, recent data indicate a considerable impact of muscular weakness and muscle wasting on quality of life in the years following critical illness.^7^, ^8^, ^9^ Importantly, it should be noted that muscular weakness and/or muscle wasting imposes an important burden not only on affected individuals, but also on health care systems.^1^, ^2^, ^3^, ^4^, ^7^, ^10^ In an effort to improve the care for patients with ICUAW, consensus guidelines on the diagnosis^11^ and research agenda^12^ were recently published.

Here, we summarize the available data on muscular weakness and muscle wasting in critically ill patients in the light of current and future perspectives. Further, we will provide an outlook on other clinically relevant neuromuscular dysfunctions [including ventilator‐induced diaphragmatic dysfunction (VIDD) and dysphagia] and will discuss potential overlap.

## Clinical presentation and nomenclature of muscular weakness in the critically ill

### Intensive care unit‐acquired weakness

A bedside diagnosis of symmetric muscular weakness and decreased muscular tone, typically of the lower limbs, in patients with critical illness should raise the potential differential diagnosis of ICUAW^2^, ^3^, ^4^ and is observed in at least 40% [1080/2686 patients, 95% confidence interval (CI): 38–42%] of ICU patients.^13^ Lack of compliance (e.g. caused by impaired communication or need for sedation), fluid shifts confounding physiologic testing and diagnostic imaging studies, and rapid onset/progression of the underlying disease often delay establishing of the diagnosis ICUAW. Neurological examination may indicate decreased or absent deep tendon reflexes, which are not pathognomic.^14^ Respiratory function may be impaired and present clinically as failure to wean from mechanical ventilation and potential overlap to VIDD should be considered in respective cases.^15^

**Figure 1:**
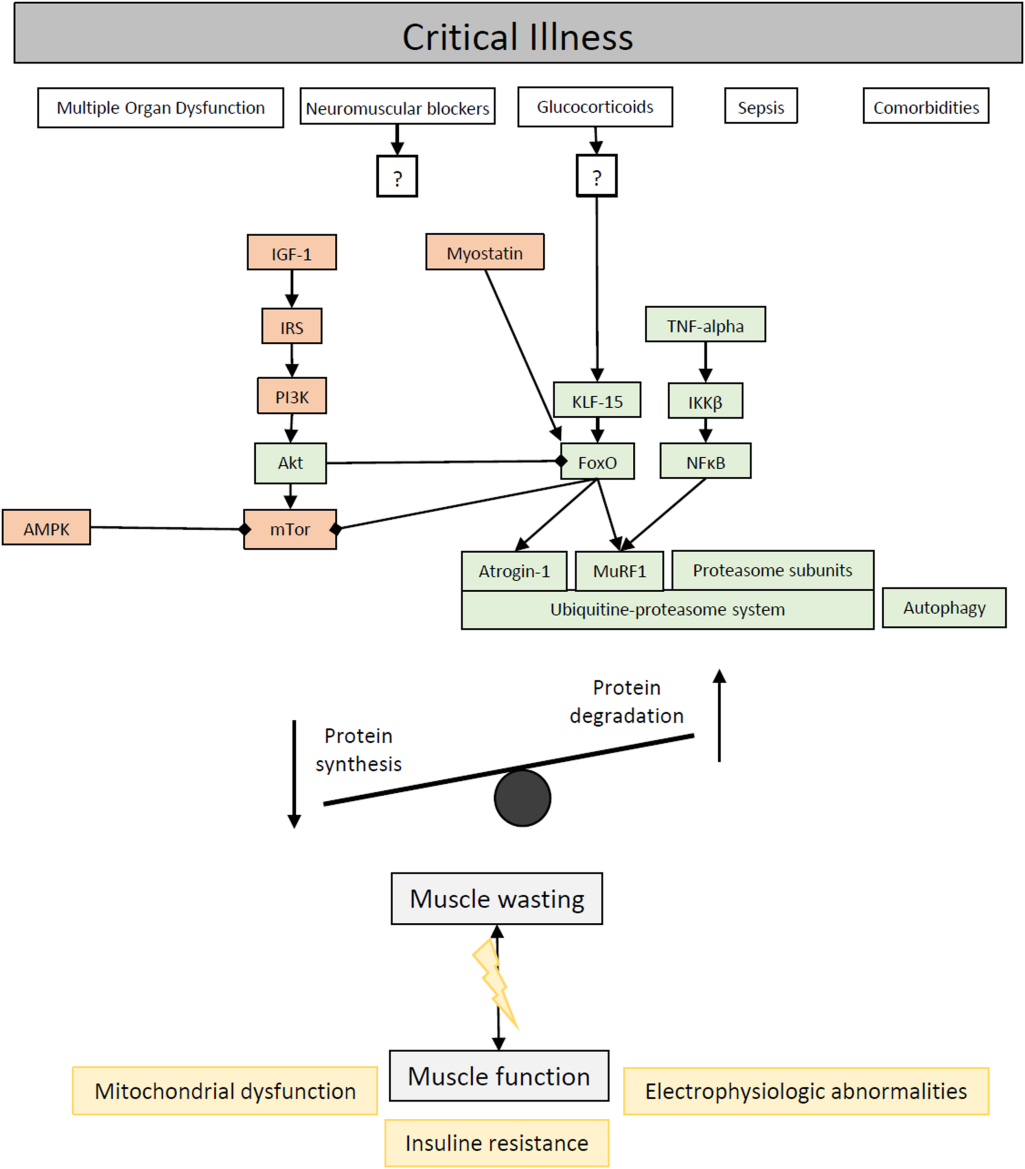
Risk factors and molecular mechanisms for muscle atrophy and muscle dysfunction in critically ill patients. Akt, Akt protein kinase B; AMPK, AMP‐activated protein kinase; IGF‐1, insulin growth factor‐1; IKKβ, inhibitor of nuclear factor kappa‐B kinase subunit beta; IRS, insulin receptor substrate; KLF‐15, Krüppel‐like factor‐15; mTor, mammalian target of rapamycin; MuRF1, muscle‐specific ring finger 1; NFκB, nuclear factor kappa‐light‐chain‐enhancer of activated B cells; PI3K, phosphoinositide 3‐kinase; TNF‐alpha, tumor necrosis factor alpha.

A round table conference in 2009^14^ proposed new definitions and ICUAW is now typically understood as the clinical ‘umbrella term’, which embraces the following subgroups: critical illness polyneuropathy (CIP), critical illness myopathy (CIM), and their combination—critical illness polyneuromyopathy (CIPM). Importantly, for differentiation between respective subtypes, electrophysiological studies [e.g. nerve conduction studies, electromyography (EMG)] and muscle biopsies are required (also refer to *Figure* 1).

### Critical illness polyneuropathy

Critical illness polyneuropathy is an acute and acquired polyneuropathy characterized by length‐dependent axonal damage.^2^, ^3^, ^4^, ^16^ Typically, thick myelinated sensory and motor fibres are affected, predominantly determining the clinical phenotype. For a definite diagnosis of CIP, published diagnostic criteria demand that patients fulfil the diagnostic criteria of ICUAW and show (in addition) typical electrophysiological evidence of an axonal motor and sensory polyneuropathy in the absence of a neuromuscular transmission deficit.^17^ If muscle strength assessment is not available, the diagnosis of *probable* CIP can be based on electrophysiological findings only. A nerve biopsy is not mandatory for the respective diagnosis. Some reports from investigations of skin biopsies demonstrate that small fibres (i.e. sympathetic fibres and C‐fibres) can also undergo degeneration in CIP patients^18^, ^19^ and may explain why patients with CIP sometimes show typical symptoms of small fibre neuropathy in the subacute and chronic phase following critical illness. Currently, it is unknown whether occurrence of CIP and acute small fibre neuropathy is linked and/or whether these two entities share common pathophysiological mechanisms.

### Critical illness myopathy

Critical illness myopathy is an acute and acquired primary myopathy.^20^, ^21^, ^22^, ^23^, ^24^ Definite diagnosis of CIM is based on a multimodal approach.^17^ Comparable with CIP, the diagnostic criteria of ICUAW have to be fulfilled. Additionally, electrophysiological studies consisting of nerve conduction studies and needle EMG are required. A neuromuscular transmission deficit needs to be ruled out using repetitive nerve stimulation. Ultimately, a muscle biopsy showing primary myopathy with myosin loss (and potential muscle cell necrosis) is needed for definite diagnosis; otherwise, only ‘probable’ CIM can be established. CIM often co‐exists with CIP, and this is referred to as CIPM. Differential diagnosis can be challenging and may require extensive electrophysiological investigation.

### Muscle wasting in critical illness

In 1892, Sir William Osler reported a ‘rapid loss of flesh’ in patients with severe infections.^25^ Today, it seems well established that muscle wasting constitutes a frequent complication in critical illness^10^ and may be most prevalent in chronic critical illness (i.e. patients with prolonged ICU length of stay). However, it seems important to note that muscular wasting constitutes a separate disease entity. Whereas ICUAW is often associated with muscle wasting, muscle wasting does not per se imply the presence of a neuromuscular disorder.^3^ Importantly, resulting muscle strength depends both on total muscle mass and force‐generating capacity (i.e. force per cross‐sectional area), which is, if reduced, a key feature in ICUAW but not necessarily in muscle wasting.^3^, ^26^

## Additional clinical presentations of neuromuscular weakness on the intensive care unit

### Ventilator‐induced diaphragmatic dysfunction

Ventilator‐induced diaphragmatic dysfunction is observed in up to 80% of ICUAW patients.^27^ VIDD can be noted in ICU patients after prolonged controlled mechanical ventilation and is determined by a rapid loss in force‐generating diaphragmatic capacity that may also affect additional respiratory muscles (e.g. the intercostal musculature).^15^, ^24^ However, VIDD can be observed as early as after a few hours of mechanical ventilation in humans and VIDD intricates with, for example, self‐induced and sepsis‐associated diaphragmatic dysfunction.^15^ As shown and discussed almost 20 years ago,^28^ VIDD may not only be considered part of weaning failure when all other aetiologies are ruled out, but should also be considered as a cause of weaning difficulty intricated with others causes (e.g. fluid overload, atelectasis). Clinically, VIDD often presents as failure to wean from mechanical ventilation despite sustained clinical efforts,^15^ and, in a first step, other underlying reasons (e.g. electrolyte abnormality or prolonged neuromuscular blockade) should be excluded. However, diaphragmatic activity can often not be assessed easily, which may hamper an early diagnosis. Clinical assessment of patients with suspected VIDD typically involves ultrasonic assessment of diaphragmatic motion (diaphragmatic dome excursion) upon spontaneous breathing trials and/or measurement of the diaphragmatic thickening fraction.^15^ Further, diaphragmatic activity can be assessed by measurement of transdiaphragmatic pressures using an oesophageal probe, or via (invasive) analysis of diaphragmatic electrical activity^15^ and phrenic nerve conduction studies.^29^, ^30^

### Dysphagia

The clinical presentation of oropharyngeal dysphagia (OD) embraces drooling from the mouth, coughing following drinking/eating (and/or silent aspiration), and clinically obvious tracheal/pulmonary aspiration.^31^ The neuromuscular process of swallowing is particularly complex, and exact underlying pathomechanisms leading to dysphagia in critical illness remain incompletely understood.^31^, ^32^ Recent data show that impaired swallowing can often be observed after mechanical ventilation in general ICU populations with up to one out of six patients with emergency admission likely affected.^33^ At ICU discharge, 10.3% (*n* = 96/933) of mixed medical–surgical ICU survivors were reported to have confirmed dysphagia and 60.4% (*n* = 58/96) of affected patients remained dysphagia positive until hospital discharge.^33^ Few studies are available on potential underlying risk factors in critically ill patients, and data show that risk factors for dysphagia and ICUAW may (at least partially) overlap.^34^ In brief, proposed key independent risk factors for dysphagia following mechanical ventilation are baseline neurological disease [odds ratio (OR) 4.45, 95% CI: 2.74–7.24, *P* < 0.01], emergency admission (OR 2.04, 95% CI: 1.15–3.59, *P* < 0.01), days on mechanical ventilation (OR 1.19, 95% CI: 1.06–1.34, *P* < 0.01), days on renal replacement therapy (OR 1.1, 95% CI: 1–1.23, *P* = 0.03), and disease severity (Acute Physiology and Chronic Health Evaluation II score within first 24 h; OR 1.03, 95% CI: 0.99–1.07, *P* < 0.01).^34^

Clinically, OD requires multidisciplinary efforts and should be systematically screened for in ICU patients at risk.^32^, ^35^, ^36^, ^37^ Reports demonstrate that awareness for dysphagia can be improved.^38^, ^39^, ^40^ In patients with undetected OD, apparent or silent aspiration may prolong ICU/hospital stay, require more ICU resources (including financial resources), and may increase morbidity and mortality.^31^, ^33^, ^35^ Further, dysphagia positivity was shown to independently predict 28‐day and 90‐day mortality (90‐day univariate hazard ratio: 3.74; 95% CI, 2.01–6.95; *P* < 0.001) and associated with a 9.2% excess (all‐cause) mortality rate.^33^

## Diagnostic approaches to generalized muscular weakness on the intensive care unit

### Clinical

Peripheral muscular weakness should be quantified by use of the Medical Research Council (MRC) sum score (MRC‐SS). The MRC‐SS includes manual assessment of three functional muscle groups on both upper extremities (shoulder abduction, elbow flexion, and wrist extension) and lower extremities (hip flexion, knee extension, and ankle dorsiflexion). Muscle strength is quantified from 0 (no movement observed) to 5 (normal contraction against full resistance). ICUAW is diagnosed via an MRC‐SS < 48, which reflects an average MRC score of <4 (antigravity strength) and as it is a diagnosis of exclusion if no other plausible aetiology for the weakness other than critical illness itself is present.^3^, ^11^, ^41^, ^42^ Furthermore, dominant‐hand dynamometry results of <11 kg force for men and <7 kg force for women may be used to identify ICUAW in previously healthy individuals.^2^, ^3^, ^4^

From a clinical perspective, a key challenge may be that the physical examination of critically ill patients on the ICU is often impeded, for example, by pre‐existing neuromuscular disease, reduced patient cooperativeness, partial sedation, prolonged neuromuscular blockade (which would typically involve cranial nerve‐innervated muscles), and/or presence of delirium, which may be particularly relevant for sensory testing as well as assessment of the MRC‐SS, and was thus shown to have a high interobserver variability.^43^, ^44^, ^45^ Importantly, in individuals incapable of voluntary contraction, electrophysiological studies would be required for diagnosis.

However, clinicians have to bear in mind that the diagnostic criteria for ICUAW as well as the clinical findings in CIP and CIM are not specific. Respective muscular weakness can also be encountered in other diseases including Guillain–Barré syndrome, myasthenia gravis, myositis, and others.^46^

Clinical findings in patients with CIP and CIM typically overlap. Deficits due to dysfunction of thick myelinated sensory nerve fibres can help to differentiate between these two entities and are only found in CIP and CIPM. In addition, one should bear in mind that deficits associated with small fibre dysfunction including temperature, pain, and sweat disturbance can be observed in CIP and CIPM.^18^ Motor deficits are hallmarks of both CIP and CIM. In CIP, flaccid muscle paresis is typically symmetrical, most prominent in distal limb muscle groups, and affects the lower extremities more. Facial muscles are spared. Distal deep tendon reflexes are reduced or abolished. Clinical motor findings in CIM resemble findings in CIP and are typically symmetrical, flaccid with facial muscles spared. However, in contrast to CIP, proximal muscle groups are more often affected than distal muscles and deep tendon reflexes are reduced and only rarely abolished.

### Electrophysiological testing

Typical electrophysiological studies in CIP and CIM consist of motor and sensory nerve conduction studies along with needle EMG. Repetitive stimulation of a motor nerve at 5 Hz is required to exclude neuromuscular transmission defects. In CIP, motor nerve conduction studies reveal reduced amplitudes of compound muscle action potentials (CMAPs) with normal distal motor latencies and normal nerve conduction velocities for stimulation of proximal nerve segments. F waves show normal latencies but are often missing. Sensory nerve conduction studies show reduced amplitudes of sensory action potentials or absent sensory action potentials. Sensory nerve conduction velocities are normal. Needle EMG typically shows signs of denervation with spontaneous activity and decreased recruitment.

In CIM, evoked CMAPs in motor nerve conduction studies are of low amplitudes (<80% of lower normal limit) with maybe increased duration due to a large variability in muscle fibre conduction velocities. Nerve conduction velocities and distal motor latencies are normal. Sensory nerve conduction studies are without pathological findings. Quantitative needle EMG reveals short duration and low amplitude polyphasic motor unit potentials with early or normal recruitment. Fibrillation potentials and positive sharp waves can be present. Comparison of CMAPs evoked by direct muscle stimulation and CMAPs elicited by stimulating the innervating nerve can help to distinguish between CIP from CIM.^47^ Two prospective multicentre studies indicated that a simplified electrophysiological screening limited to a unilateral motor nerve conduction study of the peroneal nerve has a sensitivity/specificity of 100% and 85%, respectively, for detection of CIP and/or CIM.^48^, ^49^

Recently, in CIM and CIP, more complex electrophysiological techniques were applied to detect in vivo changes of muscle and nerve membrane potentials. Muscle membrane properties can be indirectly assessed by recording multifibre velocity recovery cycles.^50^ In patients with probable CIM, it was inferred that muscle fibres were depolarized and/or that sodium channel inactivation was increased.^51^ This is in line with earlier reports measuring absolute muscle membrane potential in critically ill patients, which showed prominent depolarization of muscle resting membrane potentials.^52^ By repetitively recording muscle velocity recovery cycles in a porcine model of sepsis, it was shown that muscle membrane dysfunction can be observed within 6 h of experimental sepsis.^53^ Furthermore, using the same model, development of membrane alterations correlated with applied vasopressor (norepinephrine) dose, thus likely indicating impaired microcirculation.^54^ Nerve excitability testing is a non‐invasive approach to investigate the pathophysiology of peripheral nerve disorders by determining the electrical properties of the nerve membrane at the site of stimulation. In patients with established CIP, motor axons were shown depolarized.^55^ Membrane depolarization was associated with raised extracellular potassium levels in patients with kidney dysfunction. Furthermore, voltage‐gated sodium channel dysfunction was shown as a characteristic feature of CIP.^21^

### Histology

A significant decrease in myocyte cross‐sectional area is evident as early as Day 5 after ICU admission and persists up to 6 months after ICU discharge.^56^, ^57^ The extent of muscle atrophy correlates with the severity of illness and ICU length of stay.^10^ Data show that type II muscle fibres are mostly affected with an average rate of −4% per day during the early phase of critical illness, while fibre type distribution remains unaffected.^10^, ^57^, ^58^, ^59^, ^60^, ^61^, ^62^, ^63^ Signs for denervation atrophy (e.g. fibre type unspecific atrophy, fibre type grouping, target fibres, and atrophy of central nuclei) can be exclusively observed in CIP, but not CIM.^16^, ^41^, ^59^, ^64^, ^65^ Even though muscle necrosis is described frequently and mostly in conjunction with macrophagocytosis, it should not be viewed as pathognomic for CIM/ICUAW.^10^, ^60^, ^66^, ^67^ Besides type II fibre atrophy, selective loss of myosin filaments is characteristic for CIM.^68^, ^69^ Few reports further indicate an accumulation of both interstitial tissue and fat.^59^

Further, severe infections and sepsis are key risk factors for ICUAW.^3^ Nonetheless, results regarding inflammatory muscular infiltrates of respective patients are conflicting.^57^, ^67^ The atrophy characteristics observed during ICUAW may overlap with histological changes induced by glucocorticoids and/or neuromuscular blockers; however, they are likely not the key trigger.^70^, ^71^, ^72^ This seems underlined by the fact that corticosteroids were also shown to mediate partly protective effects if blood glucose levels are controlled.^73^ Additionally, CIP not only manifests histologically in skeletal muscles, but also in nerve tissues, and while weakness is usually aggravated proximally, the histological manifestations can often be observed to a larger extent distally.^41^, ^65^ Characteristic findings include axonal degeneration and loss of myelinated fibres in peripheral motor and sensory nerves.^16^, ^64^, ^65^ As mentioned, decreased intraepidermal nerve fibre density can also be observed in some CIP patients.^74^ Further, central nervous system involvement (including chromatolysis of the anterior horn cells and loss of dorsal root ganglion) was reported in CIP.^65^

### Electron microscopy

**Figure 2:**
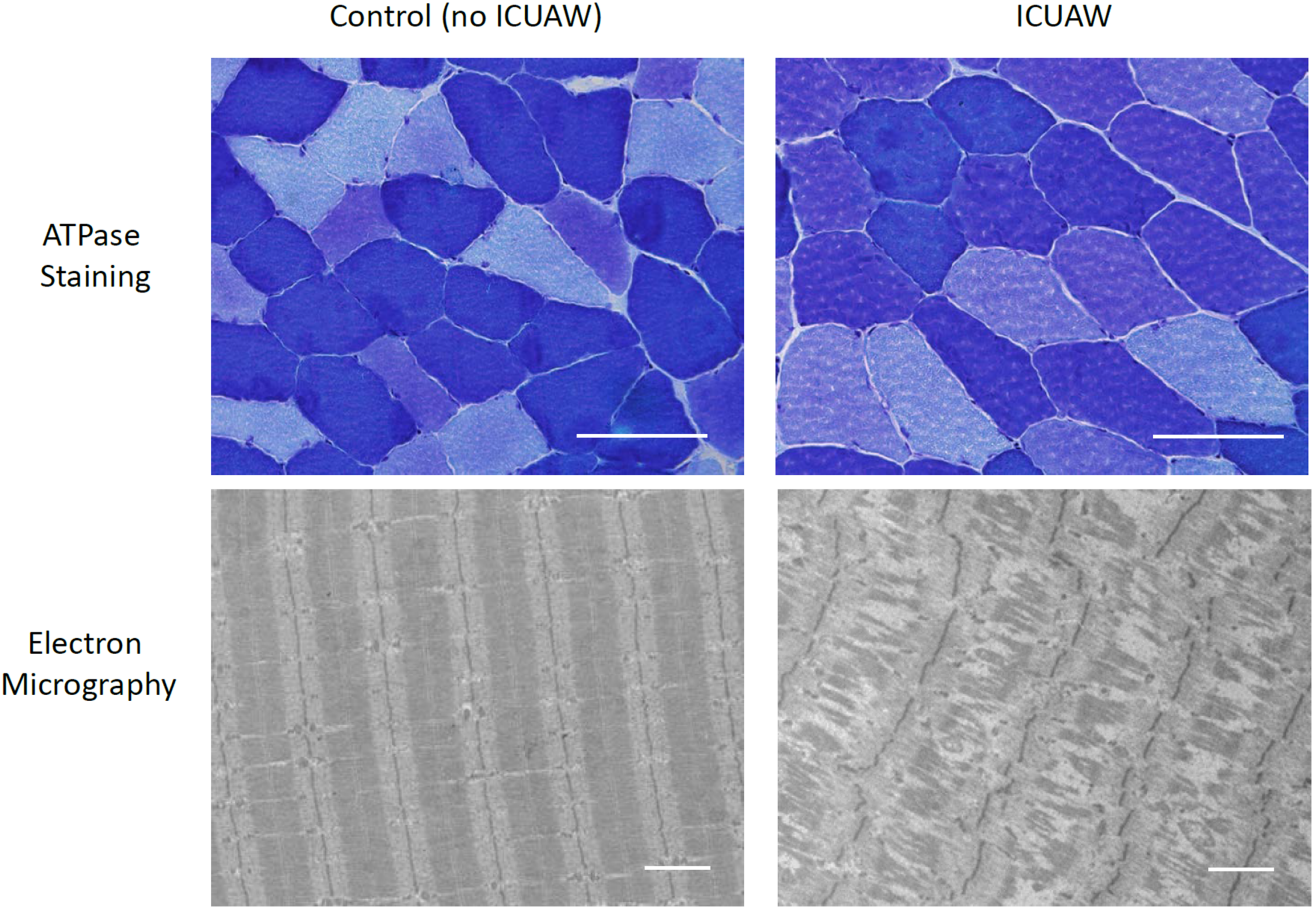
ATPase stained histologic sections and representative electron micrographs of critically ill patients with and without intensive care unit acquired weakness. Fiber types are differentiated by color (dark blue = I, intermediate blue = IIb, light blue = IIa). Scale bar indicates 100 μm (ATPase stained histologic sections) and 2 μm (electron micrographs).

The preferential loss of myosin filaments can be visualized through electron microscopy^58^, ^59^, ^68^, ^69^, ^75^ (*Figure* 2). During the early phase of critical illness, a loss of myosin filaments with preserved ultrastructure of sarcomeres is typically observed, which is lost in later stages.^20^, ^56^, ^57^, ^76^ For survivors of critical illness, a regeneration of sarcomeric ultrastructure can be observed at about 6 months after ICU discharge.^56^ Pathologic processes during critical illness affect both contractile filaments and mitochondria. In electron micrographs, swelling of mitochondria was observed early during critical illness, likely indicating mitochondrial dysfunction.^57^, ^66^ Moreover, reduced mitochondrial content and density is observed, which typically recovers until about 6 months after ICU discharge.^56^

## The pathophysiology of muscular weakness in critical illness

### Risk factors for intensive care unit‐acquired weakness

Data show that a high disease severity, number of both dysfunctional organs and of comorbidities, increases the risk for ICUAW.^23^, ^41^, ^77^, ^78^, ^79^, ^80^, ^81^, ^82^, ^83^ On top of that, patients with bacteremia/sepsis seem at a particularly high risk.^3^, ^77^, ^79^, ^80^, ^81^, ^83^, ^84^ Evidence suggests that persistent inflammation in patients with multiple organ dysfunction after acute pro‐inflammatory‐driven critical illness (e.g. sepsis, trauma) is strongly associated with end‐organ muscle inflammation, acute muscle wasting, and poor long‐term functional outcomes,^3^, ^10^, ^85^ which may be of particular importance in chronic critical illness.^86^

Although glucose levels and insulin therapy were discussed controversially, it appears that reduced serum glucose levels and higher insulin levels likely have protective effects.^23^, ^82^, ^84^ Neuromuscular blockers were also proposed as risk factors for ICUAW. However, a recent meta‐analysis on studies with a low risk of bias identified that they likely do not increase the risk.^87^ Corticosteroids were implicated early as a potential culprit in the development of ICUAW. While an association was shown by a recent meta‐analysis in the general ICU population, the findings did not extend to the subgroup of patients with sepsis. Some authors thus recommend to limit the use to low‐dose and short‐term usage in specific patient cohorts, but the discussion is overall still controversial^88^, ^89^ (please also refer to *Figure* 1).

### Decreased protein synthesis

Intensive care unit‐acquired weakness‐associated muscle atrophy results partly from decreased protein synthesis. Fractional protein synthesis rates measured via leucine incorporation are depressed on the first day after ICU admission.^10^ This is underlined by depressed mRNA expression levels for myosin heavy chains.^57^, ^71^, ^81^ A major pathway involved in muscle protein synthesis is the IGF1–PI3K–Akt/PKB–mTOR pathway.^90^ During critical illness, components of this pathway are considerably down‐regulated in patients with CIM,^91^ while Akt is up‐regulated on both transcriptional and translational levels and phosphorylated forms are more abundant in muscle biopsy specimens,^91^ which indicates that this pathway is only partially intact. Akt is physiologically able to suppress muscle protein degradation through phosphorylation of forkhead box protein (Fox)O.^92^, ^93^, ^94^ Vice versa, FoxO can suppress muscle protein synthesis through mammalian target of rapamycin (mTOR) via sestrin and by upregulation of 4E‐BP1.^95^, ^96^ On a transcriptional level, we observed increased levels of mTOR and FoxO, but the mTOR increase changes to a decrease on a translational level, hinting towards a disrupted pathway at that point.^91^ Furthermore, during sepsis, the eukaryotic initiation factor 4E (eIF4E) forms an inactive complex, which induces diminished translational activity.^22^ AMPK (a potential factor to hamper protein synthesis) was not increased and its phosphorylated form decreased.^91^ It thus seems that AMPK may therefore not be a major hindering factor for protein synthesis. Further investigations to determine exact mechanisms behind decreased protein syntheses are required.

### Increased protein degradation

As outlined above, muscle atrophy is characteristic for ICUAW with a loss in myocyte cross‐sectional area accompanied by reduced protein content for slow and fast myosin on Days 5 and 15 after ICU admission.^57^ The preferential myosin loss observed in histological staining and electron microscopy can also be observed on a protein level through a decreased myosin/actin ratio.^68^, ^69^ In fact, muscle protein degradation starts early during the course of the disease reflected by a shift in protein homeostasis towards breakdown on the first day after ICU admission,^10^ which is mediated by two key systems: the ubiquitin–proteasome pathway and autophagy.

### Ubiquitine–proteasome system

The ubiquitin–proteasome system plays a central role in ICUAW‐associated muscle atrophy. During early critical illness, FoxO1 and FoxO3 expression is induced.^57^, ^91^ While both factors were shown relevant for development of muscle atrophy, FoxO3 also directly induces atrogin‐1.^92^, ^97^ The induction of both members of the FoxO transcription factor family is reflected in the up‐regulation of muscle ring finger (MuRF)1 protein, atrogin‐1 mRNA expression, and respective proteins.^57^, ^61^ MuRF1 and atrogin‐1 are E3‐ligases that are considered to play key roles during muscle atrophy.^57^, ^61^, ^98^ Moreover, mRNA expression of proteasome subunits and ubiquitination of respective proteins is increased^20^, ^57^, ^58^, ^61^ and increased 20S‐proteasome activity can be observed up to 6 months after ICU discharge.^56^ MuRF1‐mediated muscle protein degradation was also shown to be activated by NFκB, which in turn is disinhibited through tumour necrosis factor (TNF)‐alpha via IKKβ.^99^ Whereas TNF‐alpha plasma levels are typically increased at admission in critically ill patients, its mRNA expression in muscle biopsy specimens on ICU Days 5 or 15 is not increased.^100^, ^101^ However, it should be noted that muscle biopsy specimens only allow to evaluate one given point in time of critical illness and the expression of respective cytokines may undergo rapid changes over time. Thus, it cannot be conclusively said whether, for example, TNF‐alpha contributes to development of ICUAW‐associated muscle atrophy.^102^, ^103^, ^104^

Glucocorticoids, whose involvement is still discussed controversially, are known to induce muscle atrophy via MuRF1 and atrogin‐1, which are again activated via KLF‐15 and FoxO.^98^, ^105^, ^106^ Myostatin regulates skeletal muscle mass and its lack leads to muscle hypertrophy and hyperplasia. Myostatin overexpression promotes loss of muscle mass and cachexia via the ubiquitine–proteasome pathway along FoxO1, atrogin‐1, and MuRF1.^107^, ^108^, ^109^ While induction of myostatin was suspected to be involved in ICUAW‐associated muscle atrophy, this could not be confirmed in critically ill patients.^10^

### Autophagy

Autophagy can be subdivided into three different mechanisms: (i) chaperon‐mediated autophagy, (ii) microautophagy, and (iii) macroautophagy (which is referred to in this passage). Autophagy is crucial for maintenance of muscle mass and integrity.^110^ Thus, activation and suppression of autophagy need to be well balanced as its increased activity induces muscle atrophy, while its decreased activity was suspected to play an important role in some myopathies.^111^, ^112^, ^113^

Critical illness can be considered a state of stress with increased damaged proteins and organelles due to, for example, oxidative damage. It was thus speculated that increased autophagy would be required for clearance of damaged proteins/organelles in an effort to maintain cellular integrity and function. Recent data show that during critical illness, insufficient activation of autophagy can be observed, which is reflected in vacuolization of both myofibers and central nuclei, as well as accumulation of p62 and ubiquitinated proteins.^114^ It was subsequently shown that the extent to which autophagy could be activated in critically ill patients (as reflected by the LC3II:LC3I ratio) was protective in regard to development of muscle weakness.^81^

### Glucose metabolism

Hyperglycemia is common during critical illness and mostly results from both increased hepatic glucose liberation and reduced peripheral muscular glucose uptake due to insulin resistance.^115^, ^116^, ^117^ Such insulin resistance is also reflected by a reduced insulin sensitivity index and a lack of muscular metabolic responses during hyperinsulinemic–euglycemic clamp.^91^ Reduction of the insulin sensitivity index was shown pronounced in CIM patients when compared with non‐CIM patients.^91^ Diminished metabolic responses to insulin are paralleled by a decreased relative mRNA expression of SLC2A4 and impaired translocation of glucose transporter (GLUT) 4 to the sarcolemmal membrane, both of which are aggravated in CIM as compared with non‐CIM patients.^91^ Interestingly, the insulin receptor pathway involved in GLUT4 translocation appears intact up to the level of Akt phophorylation because an increase in phosphorylated Akt can be observed. Other downstream components of the insulin‐dependent GLUT4 translocation pathway (e.g. AS160, Rab protein) were not implicated in ICUAW so far.^118^ Future investigations are necessary to elucidate the pathomechanisms behind the observed effects of insulin therapy for ICUAW.

### Channelopathy

An early feature of CIM is a non‐excitable muscle membrane determined via a direct muscle stimulation CMAP of <3 mV.^119^ Abnormal excitability of muscle membranes might thus result from decreased voltage‐dependent sodium channel availability due to inactivation,^51^ which could be due to circulating factors (e.g. endotoxins).^51^, ^120^ In agreement, motor neuron sodium channel inactivation was observed in CIP.^21^ Moreover, axonal membrane depolarization is observed during CIP, which is likely caused by increased extracellular potassium levels and/or hypoxia.^55^

## Therapeutic strategies in neuromuscular weakness of intensive care unit patients

### General strategies: preventive concepts and early recognition

Evidence‐based (causal) therapies for ICUAW, CIP/CIM/CIPM, VIDD, and/ or swallowing disorders are currently unavailable, which underlines the importance of preventive strategies and/or strategies for early recognition of patients at risk. This may (partly) allow for avoidance of risk factors and may support the prevention of worsening ICUAW. In this regard, prevention and aggressive treatment of severe infections/sepsis and associated shock states should be aimed for.^1^, ^2^, ^3^ Prevention of risk factors further embraces avoidance of prolonged bed rest, ‘over’‐analogosedation, and/or neuromuscular blockade whenever possible. In summary, preventive strategies include minimization of risk factors as well as sedation/neuromuscular blockade. Development of novel preventive and/or therapeutic strategies in affected patients may also be challenged by the acuteness and severity of the underlying (rather heterogeneous) diseases.

### Early mobilization

On the ICU, multidisciplinary efforts should aim to reduce the duration of immobilization by early start of physical therapy (e.g. with in‐bed cycling^121^). Although direct evidence is sparse, ICU length of stay, for example, can likely be reduced by such early mobilization concepts. Further, few data demonstrate that early physical therapy may improve muscular strength and ICU outcomes.^122^ Despite obvious clinical benefits of early mobilization, however, in a recent Cochrane review,^123^ there was insufficient evidence to prove that physiotherapeutic measures would shorten the time of rehabilitation from critical illness. Despite this, to prevent or treat VIDD, patients should be weaned from controlled mechanical ventilation as early as possible and adapted to spontaneous breathing capacity.^15^ With regard to electrostimulation of the diaphragm, or of the swallowing apparatus, no direct evidence is currently available. Large‐scale clinical trials (e.g. on neuromuscular dysphagia) are currently performed.^124^ In peripheral muscle groups, neuromuscular electrical stimulation was shown to preserve muscle mass and prevent muscle atrophy in critically ill patients. It was nevertheless not able to diminish the functional decline observed in ICUAW.^125^, ^126^

### Nutritional interventions

Data indicate that an intensive insulin therapy (that would target blood glucose levels to 80–110 mg/dL) might reduce the incidence of ICUAW. Importantly, however, such intensive insulin therapy was shown to increase the number of hypoglycemic episodes and may actually worsen clinical outcomes in general ICU cohorts.^127^, ^128^, ^129^ Thus, although euglycemia (treatment of ICU patients using insulin at blood glucose levels > 180 mg/dL) should likely be generally aimed for, an intensive insulin therapy is not advised. Further, a catabolic state is typically observed in (the early phase of) critical illness and better understanding of metabolic changes could theoretically provide novel therapeutic avenues for the treatment of ICU patients.^130^ Previously, early parenteral nutrition was proposed to counteract critical illness‐induced catabolism, but recent data show that the early catabolic phase of critical illness can likely not be averted by artificial nutrition and that this may actually lead to adverse clinical outcomes, including higher ICUAW incidence.^10^ Thus, although nutritional interventions appear appealing, direct evidence is missing warranting further research.

### Intensive care unit‐acquired weakness‐associated complications

Prolonged bed rest in critically ill patients typically increases the risk for additional comorbidities. This includes venous thrombosis, pressure ulcers, atelectasis, and mood disorders including anxiety and depression. Prophylaxis for thrombosis and pressure ulcers, as well as symptomatic therapy of complications, is advised.

## Outcome assessment

### Muscle strength: Medical Research Council score

Manual muscle strength testing via the MRC score is the current guideline recommendation for diagnosing ICUAW.^11^ Multiple studies showed a high interrater reliability of the MRC‐SS^6^, ^43^, ^44^, ^45^, ^131^, ^132^ with differences between scores of 4 and 5 contributing to most between‐rater incongruences.^43^ Data on the need for periodical rater training are lacking. Further, muscle strength at ICU discharge is directly associated with mortality 5 years after discharge (hazard ratio 0.946, 95% CI: 0.928–0.968 per point increase in the MRC score, *P* = 0.001).^133^

### Hand grip strength

In line with data on manual muscle testing, hand grip strength has an acceptable interrater reliability in ICUAW.^43^, ^132^, ^134^ Its sensitivity and specificity for ICUAW are above 80% for the cutoffs <11 kg (in men) and <7 kg (in women).^6^

### Muscle function: the 6‐min walking distance test

The 6‐min walking distance test (6MWD) measures muscle function and correlates both with physical function and health‐related quality of life.^135^, ^136^, ^137^ Besides good convergent validity, the 6MWD has acceptable discriminant validity as it does not correlate with mental health‐related quality of life.^137^ As changes over time may be considered more important than absolute values, the 6MWD may especially be useful during (clinical) follow‐up.^137^ Limitations embrace the fact that repetitive testing and track length impact on the absolute 6MWD^135^ and that many ICU patients would be unable to perform the test.

### Short form health questionnaire (SF)‐36

The SF‐36 is commonly used for evaluation of health‐related quality of life after critical illness. It has sufficient reliability and validity in survivors of critical illness.^138^ Nevertheless, data (specifically in ICUAW) are scarce and of low quality.^139^

### Long‐term disability

Investigations on long‐term outcomes from critical illness differ largely regarding the assessment tools applied,^140^ resulting in considerable data heterogeneity. This limits data comparability and standardized outcome data sets are currently awaited.^141^, ^142^, ^143^ Nevertheless, although physical function is regarded highly important after critical illness, the actual physical status and generalized fatigue may be key reasons to prevent follow‐up assessment(s) and return to work.^144^, ^145^, ^146^ In summary, available data regarding physical performance are of poor to fair quality and further research is warranted.^139^

## Economic burden

Intensive care unit‐acquired weakness has a major economic impact. ICUAW was shown to prolong mechanical ventilation and ICU and hospital length of stay, and it prevents physical recovery (i.e. timely rehabilitation).^8^, ^9^, ^147^ Despite major individual limitations, this also has considerable impact on public health care systems and on society.

### Short‐term/initial hospitalization

Health care resources are largely affected by ICUAW. Early data from 1996 show that patients with neuromuscular weakness have significantly increased overall treatment costs.^148^ Hermans *et al*. further demonstrate significantly higher treatment costs on the ICU, whereas post‐ICU (ward) costs were rather comparable with patients without ICUAW.^147^ Both investigations matched patients and controls for ICUAW risk factors.

### Post‐discharge/long‐term

Patients surviving critical illness face serious economic challenges most likely due to the ‘post‐intensive care syndrome’. Only about two thirds of patients that were working before critical illness return to work within a 12‐month post‐discharge period. Further, the cumulative loss in annual earnings is about 60% of previous income.^149^ Five years after ICU discharge, only about 77% of patients returned to work.^8^ Return to work is often hampered by depression, post‐traumatic stress, persisting muscle weakness and fatigue, and cognitive disability.^145^ The negative economic impact is considerable with half of affected families making major lifestyle adjustments in order to provide care.^150^ Further, after discharge of patients with ICUAW, the economic burden for society is considerable and patients are readmitted to hospitals more frequently.^145^, ^151^ However, data on the specific economic impact of ICUAW post‐discharge are scarce and further research is required.

## Conclusions and future perspectives

In conclusion, loss of muscle mass and/or presence of muscle weakness represents a key medical challenge that affects a large number of patients on the ICU. Survivors of (prolonged) critical illness are often affected by the ‘post‐intensive care syndrome’, which is mainly characterized by long‐term physical and mental disability.

Despite the ‘classic’ presentation of symmetric peripheral weakness of limb muscles as in ICUAW, diaphragmatic muscular dysfunction and other neuromuscular dysfunctions including swallowing disorders were of major importance regarding morbidity and mortality of affected ICU patients. Importantly, evidence‐based causal therapies for ICUAW, CIP/CIM/CIPM, VIDD, and dysphagia are currently not available, which underlines the paramount importance of preventive and early diagnostic measures. Such measures include systematic screening (and thus identification) of patients at risk, avoidance of deep sedation/prolonged neuromuscular blockade, reducing length of mechanical ventilation whenever possible, promotion of early mobilization, as well as metabolic and nutritional control.

It seems that key to a better understanding of neuromuscular dysfunctions in critical illness is that there is considerable overlap regarding underlying pathomechanisms, and this understanding may open new research avenues. In the future, thorough studies investigating underlying pathomechanisms and innovative therapeutical approaches are highly warranted for critically ill patients with muscular weakness and muscle wasting.

## Conflict of interest

J.C.S. reports grants from Orion Pharma, Abbott Nutrition International, B. Braun Medical AG, CSEM AG, Edwards Lifesciences Services GmbH, Kenta Biotech Ltd, Maquet Critical Care AB, Omnicare Clinical Research AG, Nestle, Pierre Fabre Pharma AG, Pfizer, Bard Medica S.A., Abbott AG, Anandic Medical Systems, Pan Gas AG Healthcare, Bracco, Hamilton Medical AG, Fresenius Kabi, Getinge Group Maquet AG, Dräger AG, Teleflex Medical GmbH, Glaxo Smith Kline, Merck Sharp and Dohme AG, Eli Lilly and Company, Baxter, Astellas, Astra Zeneca, CSL Behring, Novartis, Covidien, Phagenesis, and Nycomed outside the submitted work. The money went into departmental funds. No personal financial gain applied. All other authors declare no conflict of interest.
